# Gene co-occurrence and its association with phage infectivity in bacterial pangenomes

**DOI:** 10.1098/rstb.2024.0070

**Published:** 2025-09-04

**Authors:** Anne Kupczok, Athina Gavriilidou, Emilian Paulitz, Lucía Guerrero-García, Franz Baumdicker

**Affiliations:** ^1^ Bioinformatics Group, Wageningen University, Wageningen, Gelderland, The Netherlands; ^2^ Department of Computational Biology, University of Lausanne, Lausanne, Vaud, Switzerland; ^3^ Swiss Institute of Bioinformatics, Lausanne, Vaud, Switzerland; ^4^ Bioinformatics Department, Institute of Biochemistry and Biology, University of Potsdam, Potsdam, Brandenburg, Germany; ^5^ Systems Biology and Mathematical Modeling Group, Max Planck Institute of Molecular Plant Physiology, Potsdam, Brandenburg Germany; ^6^ Institute for Bioinformatics and Medical Informatics (IBMI), University of Tübingen, Tübingen, Baden-Württemberg, Germany; ^7^ Cluster of Excellence 'Controlling Microbes to Fight Infections', University of Tübingen, Tübingen, Baden-Württemberg, Germany

**Keywords:** bacterial pangenomes, phage infectivity, phylogenetics, bacterial defense systems, gene presence and absence

## Abstract

Phages infect bacteria and have recently re-emerged as a promising strategy to combat bacterial infections. However, there is a lack of methods to predict whether and why a particular phage can or cannot infect a bacterial strain based on their genome sequences. Understanding the complex interactions between phages and their bacterial hosts is thus of considerable interest. We recently developed Goldfinder, a phylogenetic method to discover gene co-occurrences across bacterial pangenomes. Here, we expand Goldfinder to infer which gene presences or absences influence bacterial sensitivity to phages. By integrating a bacterial pangenome with an experimentally determined host range matrix, we infer associations between phage infectivity and the presence of accessory genes in bacterial pangenomes. The presented approach can be applied to predict bacterial genes that potentially enable phage infection, bacterial genes that prevent phage infection, and potential interactions between particular bacterial and phage accessory genes. Finally, the predicted interactions are clustered and visualized with the software Cytoscape. Here, we present a method to identify candidate genes within the pool of mobile accessory genes that may contribute to phage–host interactions. This approach will help to set up follow-up experiments and to understand the complex interactions between phages and bacteria.

This article is part of the discussion meeting issue ‘The ecology and evolution of bacterial immune systems’.

## 1. Background

Bacteriophages are viruses that infect bacteria, and they are ubiquitous in all environments. Their potential as therapeutic agents, especially in the context of rising antibiotic resistance, has renewed interest in understanding their interactions with bacterial hosts [1]. Since phages need bacteria to replicate, bacteria are under constant risk of phage infection, resulting in an evolutionary arms race. Phage infection is a multi-step process, composed of phage adsorption to a bacterial receptor, injection of phage DNA and phage replication within the bacterial cell. In response to this persistent threat, bacteria have evolved numerous strategies to defend themselves against phage infection at all these stages (i) by preventing adsorption by modifying surface receptors or capsule components; (ii) by preventing DNA injection, e.g. in superinfection exclusion systems; and (iii) by intracellular defense systems that interfere with the phage DNA or inhibit the phage life cycle, e.g. restriction modification systems or CRISPR/Cas systems [2]. In turn, phages have evolved a multitude of strategies of counterdefense, such as variability in receptor-binding proteins and diverse anti-defense mechanisms [3,4]. Thus, during phage infection, a complex network of molecular interactions determines whether a particular infection is successful for the phage or successfully defended by the bacteria, where success is clearly a matter of perspective.

It is believed that this evolutionary arms race substantially contributes to the considerable variation in gene content observed among individuals in bacterial populations. The pangenome of a population, i.e. the set of all genes present in the population, generally contains many accessory genes that are present in only some of the strains of a species [5,6]. Mobile genetic elements, such as plasmids, prophages and transposons, can be transferred horizontally between genomes and account for the large diversity observed in the accessory genome. It recently became clear that defense systems are often linked with mobile genetic elements. Owing to fitness costs of defense systems, e.g. the risk of auto-immunity, individual genomes often carry only a fraction of the defense systems found in a species [2]. For example, most defense systems found in *Pseudomonas aeruginosa* are present in <10% of the strains [7].

As a consequence of the rapid evolutionary arms race, phages are often strain-specific and the phage host range is determined by the bacterial and phage genomes [7]. Nevertheless, it is still challenging to infer the genomic components in phages and bacteria that determine the outcome of an infection.

To address this, we build upon Goldfinder, a phylogenetic method originally developed to detect gene co-occurrences within bacterial pangenomes [8]. In this study, we demonstrate how to integrate bacterial pangenomic data with matrices of phage–bacteria interactions to identify associations between phage infection patterns and bacterial genes. Additionally, in cases where the phage genomes are available, we also show how to include this information to predict potential associations between phage and bacterial genes. We show that this approach can detect relationships between infection outcomes and biologically plausible features, such as pilus assembly proteins, while providing a framework for discovering novel associations.

## 2. Results

### (a) Combining pangenomes with phage–host interaction data to infer associations between phage and bacterial genetic features

Goldfinder is a phylogenetic method designed to discover gene co-occurrences across bacterial pangenomes with several advantages compared with other existing tools [8]. To infer gene pairs that co-occur owing to co-selection or co-transfer instead of co-inheritance along the phylogeny, Goldfinder uses simulations to estimate an accurate neutral null distribution. To consider more information than gene presence/absence in the strains, Goldfinder reconstructs ancestral states and gene gains and losses along the phylogeny. This allows the frequency with which genes were co‑gained (or co‑lost) to be taken into account. Additionally, Goldfinder can reconstruct and visualize clusters of co-occurring genes.

**Figure 1.**
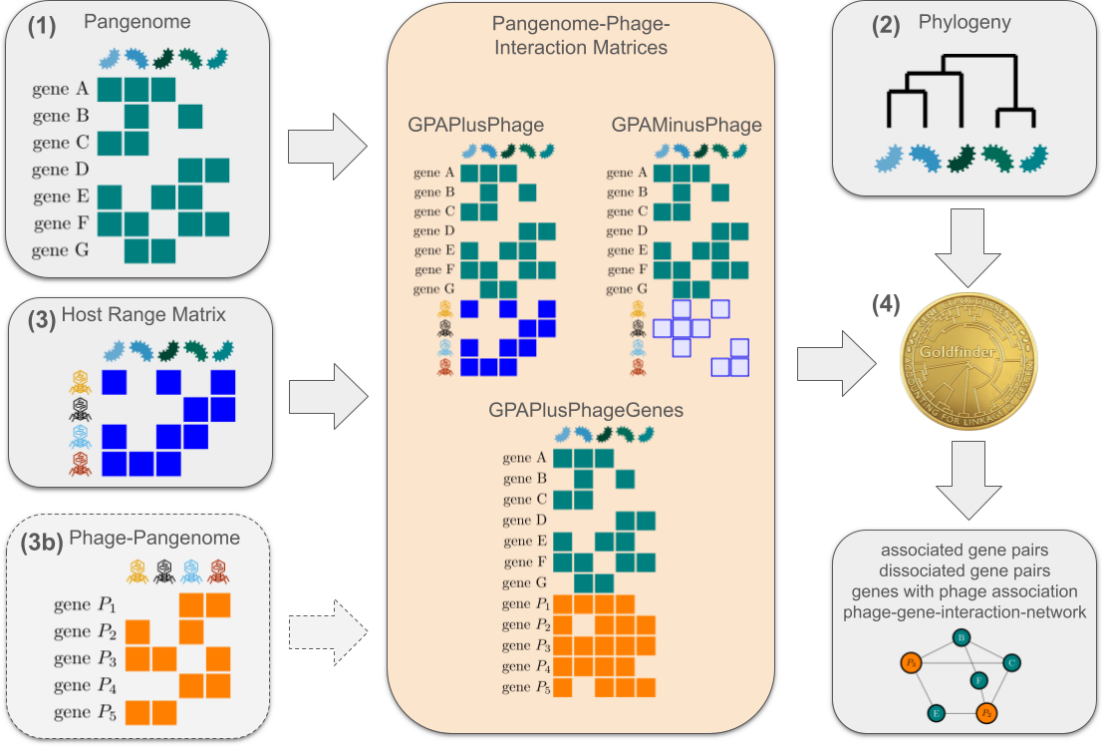
Workflow for the joint analysis of gene co-occurrence and phage host range. The required input of our method is (1) a gene presence/absence matrix representing the pangenome of the bacterial strains under consideration; (2) a phylogenetic tree for these strains; and (3) a host range matrix that indicates which phages can or cannot infect or adsorb to each bacterial strain. The two matrices are combined into two different pangenome–phage interaction matrices, which are then used alongside the phylogeny provided to the software Goldfinder (step 4). The GPAPlusPhage matrix and the GPAMinusPhage matrix can be generated from the Host Range Matrix and allow the identification of the accessory genes that enable or prevent phage infection/adsorption. To associate phage genes with bacterial genes, a phage pangenome (3b) is required as input to generate the GPAPlusPhageGenes matrix.

However, Goldfinder in its original form does not incorporate phenotypic or interaction data. To infer which gene presences or absences influence phage infection, we extended Goldfinder with a multi-step approach (figure 1):

— Construction of a bacterial pangenome using an available tool such as Panaroo [9] or panX [10]. This step will result in homologous groups and a gene presence–absence (GPA) matrix describing the strains in which each homologous group occurs.
— Reconstruction of a phylogenetic tree for the set of bacterial strains, e.g. based on the core genes, alignment using an available tool such as IQ-TREE [11].
— Combination of the GPA matrix with an experimentally determined host range matrix for a set of phages, where complete information is available on which phage strain can (and cannot) infect a bacterial strain from the pangenome. When doing so, we treat a phage’s ability (or inability) to infect a strain in the same way as the presence or absence of accessory genes. At this step, we can generate two different kinds of pangenome–phage interaction matrices: in the GPAPlusPhage matrix, a phage that can infect the strain is encoded like the presence of a gene. In contrast, in the GPAMinusPhage matrix, a phage that *cannot* infect the strain is encoded like the presence of a gene.
— Analysing the Pangenome–Phage Interaction Matrix from Step 3 with Goldfinder to infer associations between phage infection ability and bacterial accessory genes. In detail, the Goldfinder method will reconstruct for each phage whether it is able to infect the ancestral strains along the phylogenetic tree and search for GPA changes along the tree that are associated with susceptibility to this phage. For the GPAPlusPhage matrix, this approach will infer candidate genes whose presence is associated with phage infection. For the GPAMinusPhage matrix, we will infer genes whose absence is associated with phage infection, i.e. genes that have the potential to defend against phage infection.

In addition, if the phage genomes are also available, we can also construct the GPAPlusPhageGenes matrix in step 3 as follows: homologous families of phage genes are reconstructed and each phage gene family is added to the bacterial pangenome. At this step, a phage gene is encoded as present in a bacterial strain if it is present in a phage that can infect the strain. When analysing this matrix with Goldfinder in step 4, we will infer which candidate bacterial genes are present in bacterial strains that can be infected by phages that carry a particular gene.

Importantly, all GPA matrices described above contain the whole bacterial pangenome. Thus, the Goldfinder algorithm will also detect gene–gene interactions within the pangenome itself.

### (b) Visualizing the network of interactions between genes and phages

**Figure 2.**
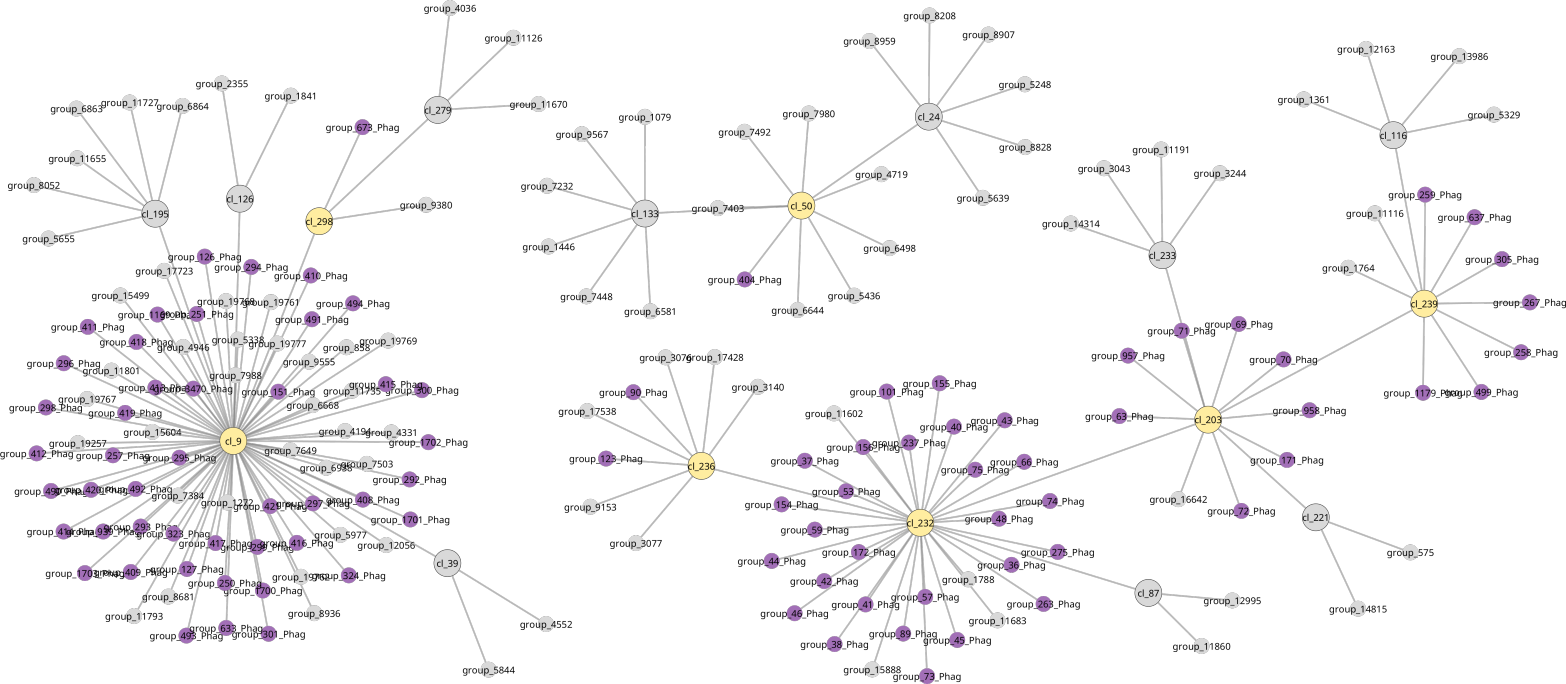
Visualization of a subset of the pangenome–phage association network in Cytoscape: small nodes represent bacterial genes (grey) or phage genes (purple), while larger nodes indicate cluster centres. Genes within the same cluster of associated genes are connected to their cluster centre. Cluster centres are coloured the same as the genes they comprise if they all belong to the same category, while ‘mixed’ cluster centres are shown in yellow. We show a subnetwork of the mixed clusters with at least one association between a phage and a bacterial gene within the cluster. We also added the connected clusters, where the number of gene–gene associations between genes of this cluster and the mixed cluster exceeds 10% of all possible gene pairs between the two clusters (see §4 for more details).

When analysing the different pangenome–phage interaction matrices with Goldfinder, we will predict a list of pairwise gene–gene and gene–phage associations and dissociations whose occurrences are not easily explained by co-inheritance along the provided phylogeny. The pairwise associations give rise to a complex interaction network. To explore this network, we provide a clustering and visualization script that generates an interactive graph that can be opened with the software Cytoscape [12]. This graph has different types of nodes: nodes that represent phages, phage genes, bacterial genes or the centre of clusters of co-occurring genes or phages. Nodes are coloured by their type and can be connected by an edge, indicating a certain type of interaction. In the example shown in figure 2, genes and phages are connected to the cluster centre nodes if they belong to the same cluster of co-occurring genes or phages. In addition, cluster centres can be connected to other cluster centres when the number of pairs of elements that co-occur and span both clusters exceeds a certain significance threshold that can be set in Cytoscape.

### (c) Example analysis of *Vibrio* strains and phages

As an example, we analysed the pangenome of 244 *Vibrio* strains and 227 phages infecting them in combination with the corresponding host range matrix [13]. When analysing the results based on the GPAPlusPhage matrix, we find 23 613 significantly associated pairs (*p* < 0.05), of which 23 449 are between bacterial accessory genes, 120 are between different phages and 44 are between 19 different phages and bacterial accessory genes. The clustering resulted in 304 clusters, of which 300 involve only bacterial genes and 4 involve both phages and bacterial genes (19 phages and 26 bacterial genes in total; table 1). Thus, we do not find associations for 208 (92%) phages in the dataset. Note that Goldfinder finds co-occurring pairs based on the provided phylogeny. Thus, for a pair to be significant, a phage and a bacterial gene must be gained and lost together multiple times along the phylogeny. However, a large part of the dataset is composed of phages infecting only a few strains, where 105 (46%) infect only one bacterium, 28 (12%) can infect two bacteria and 21 (9%) can infect three bacteria. We observe that the phages without associations can infect < 20 bacteria, while 17 of the phages with associations can infect more than 20 bacteria and 2 infect 14 bacteria. This highlights the importance of having a relatively high number of interactions per phage to detect associations. More precisely, a complex pattern of phage–bacteria interactions that is distributed among different clades of the phylogeny helps to identify associations. The selection of strains and the design of the host range analysis are thus important factors that determine which associations are detectable. The genes associated with the 19 phages include biologically relevant functions and potential phage entry points, such as pilus assembly proteins and ABC transporters. We find that one phage was associated with a transposase. Transposases are often linked to prophage elements or phage-derived genetic islands, suggesting that they may also be remnants of past phage infections. Similarly, we also found in some of the bacterial genomes a tyrosine-type phage integrase that is associated with the ability of phage 1.215.A to infect these strains. Note that some associations, such as the previous two examples, do not necessarily explain the observed patterns of susceptible strains directly, as they may also arise owing to co-occurrence patterns created by (similar) phages that infected some of the ancestors of the susceptible strains in the past.

**Table 1.** Association clusters with phage nodes detected in the *Vibrio* dataset.

cluster	gene	annotation
**GPAPlusPhage**		
85	group_9598	ribosomal protein acetyltransferase; GNAT family N-acetyltransferase
	group_13733	hypothetical protein
	group_4257	integrase; hypothetical protein
	group_9872	nuclease; NERD domain-containing protein
	group_3434	hypothetical protein
	group_12215	DJ−1/PfpI family protein; thiamine biosynthesis protein ThiJ
	group_9686	integrase; phage integrase N-terminal SAM-like domain-containing protein; tyrosine-type recombinase/integrase
	1.215 .A._Phag	
229	group_21844	transposase
	2.117.O._Phag	
239	group_4798	arginine deiminase
	group_11735	DEAD/DEAH box helicase
	group_4194	restriction endonuclease subunit M
	group_858	hypothetical protein
	group_19257	hypothetical protein
	group_19769	ferric anguibactin receptor
	group_19768	iron ABC transporter permease
	group_19767	ferric anguibactin-binding protein
	group_19762	iron ABC transporter permease
	group_19777	ABC transporter
	group_19761	ABC transporter
	1.011.O._Phag	
	1.062.O._Phag	
	1.040.O._Phag	
	1.044.O._Phag	
	1.043.O._Phag	
	2.092.O._Phag	
	1.102.O._Phag	
	1.080.O._Phag	
	1.125.O._Phag	
	1.008.O._Phag	
	1.095.O._Phag	
	1.141 .A._Phag	
	1.069.O._Phag	
	1.048.O._Phag	
	1.057.O._Phag	
	1.107.B._Phag	
291	group_18110	pilus assembly protein CpaC
	group_18109	AAA family ATPase
	group_3429	hypothetical protein
	group_307	Flp pilus assembly protein CpaB
	group_6892	hypothetical protein
	group_6789	hypothetical protein
	group_5524	hypothetical protein
	1.139.B._Phag	
**GPAMinusPhage**		
43	group_5512	DUF3187 family protein; hypothetical protein
	group_12076	transposase; IS110 family transposase; transposase
	group_10811	hypothetical protein
	1.215 .A._Phag	

For the GPAMinusPhage matrix, we found 23 549 significantly associated pairs (*p* < 0.05), of which 23 427 are between bacterial accessory genes, 120 are between different phages and 2 are between phages and bacterial accessory genes. Thus, these genes are predicted to be associated with the disability to be infected by certain phages. We find a transposase associated with phage 1.215.A and a ligand-gated channel protein associated with phage 1.125.O. The transposase is co-occurring with 11 further genes of which 2 are clustered into the same cluster (table 1). We find that the ligand-gated channel protein is not clustered with the associated phage. A possible reason for this is that the phage clusters with 15 related phages, whereas the ligand-gated channel protein clusters with 63 other bacterial genes, which separates them into distinct clusters. Nevertheless, this significant association that spans the two clusters can still correspond to a meaningful biological association.

Our approach is not limited to phage–gene associations, but we can also consider the gene–gene associations within the *Vibrio* pangenome. Typically, these results are almost identical for GPAPlusPhage and GPAMinusPhage. As an example, we identified 1066 bacterial accessory genes that are part of a defense system using DefenseFinder [14]. Goldfinder predicts six clusters of co-occurring genes that contain at least one defense system and further genes associated with defense genes, including another integrase and a ribonuclease (table 2) in cluster 195. Nevertheless, consistent with observations for *E. coli* [15], we do not find associations between phage absence and defense systems.

**Table 2.** Association clusters with defense systems predicted in the *Vibrio* dataset.

cluster	gene	annotation (defense system—DefenseFinder Gene Hit)
**GPAPlusPhage**		
192	group_10183	deoxyribonuclease HsdR (**RM**—Type I REases)
	group_9389	RM system subunit M (**RM**—Type I MTases)
195	group_11655	hypothetical protein; lecithin retinol acyltransferase family protein
	group_8052	ribonuclease HI
	group_6864	integrase; tyrosine-type recombinase/integrase
	group_6863	hypothetical protein; DUF2787 domain-containing protein
	group_5655	hypothetical protein; DUF2787 domain-containing protein
	group_11727	DNA-binding protein (**MADS**—mad1, **RM**—Type I MTases, **RM**—Type IV REases)
227	group_12104	S-adenosylhomocysteine hydrolase (**SanaTA**—SanaA)
	group_12106	pyrophosphatase (**SanaTA**—SanaT, RM—Type I MTases)
261	group_13129	ATPase (**PD-Lambda-1**—PD-Lambda-1)
	group_10215	hypothetical protein (**PD-Lambda-1**—PD-Lambda-1)
290	group_11981	hypothetical protein (**PrrC**—RM Type I REases)
	group_11561	diguanylate cyclase; DEAD/DEAH box helicase family protein (**PrrC**—RM Type I REases)
308	group_10534	ATP F0F1 synthase; hypothetical protein (**Kiwa**—KwaB_2)
	group_10168	hypothetical protein (**RM**—Type I S, **Kiwa**—KwaA)
	group_10166	restriction endonuclease subunit R (**RM**—Type I REases)
	group_9316	diguanylate cyclase; DEAD/DEAH box helicase family protein (**RM**—Type I S, **RM**—Type I MTases)

Finally, when analysing the GPAPlusPhageGenes matrix, we find significant associations (*p* < 0.05) for 174 074 pairs, of which 138 212 (79%) are between phage genes and 3003 (1.7%) are between phage genes and bacterial genes. The high number of associations between phage genes is expected since genes from the same phage are expected to show co-occurrences. In total, 14 clusters that contain 374 phage and 109 bacterial genes have been identified for the *Vibrio* dataset. Note that, of these 374 phage genes, 232 occur in the 19 phages found significant with the GPAPlusPhage matrix and 228 occur in the remaining phages, of which 86 occur in phages from both groups. Thus, even when no significant associations have been detected for a particular phage, they can still be detected for phage genes. This complex network of associated phage and bacterial genes can be explored in detail using the network visualization. A condensed visualization of a subgraph of seven clusters of this network is shown in figure 2.

## 3. Discussion

Owing to the increasing prevalence of antimicrobial resistance in bacterial pathogens, phages recently (re-)emerged as a promising strategy to combat bacterial infections [1]. However, finding phages that are specific to a particular strain is costly in terms of time and resources. Methods that predict promising candidates only from the phage and bacteria genome sequences would speed up this process. Recent advances showed that the outcome of phage–bacteria interactions can be predicted with reasonable accuracy for *Escherichia* strains and phages [15]. However, general statistical approaches that can be applied to diverse taxa are currently lacking, and the proposed approach aims to fill this gap.

Here, we present a method to detect associations between phage infectivity and the presence of accessory genes in bacterial pangenomes. The methodology builds on the Goldfinder approach and thus provides an integrative view by combining gene co-occurrences within the bacterial pangenome with phage infectivity. By using different encodings—(i) ability of phages to infect, (ii) inability of phages to infect and (iii) gene presences in phages that are able to infect—the approach allows us to infer (i) bacterial genes that enable phage infection, (ii) bacterial genes that prevent phage infection and (iii) the interactions between particular bacterial and phage accessory genes.

In addition to the associations between the bacterial pangenome and phage infectivity, Goldfinder will also infer gene–gene interactions within the pangenome itself. This is particularly relevant given the recent discovery of numerous defense systems in bacterial genomes. The functioning of some of these systems likely depends on the presence of other defense systems in the genome [16,17]. The proposed approach can be used to infer different types of associations simultaneously, for example, between different defense genes, between defense genes and genes of the genomic background, between defense genes and phages or between defense genes and phage genes. It thus has a wide scope of application, including the potential to reveal synergies between defense genes as well as to predict interactions between defense and anti-defense genes.

Furthermore, the presented method can be used to study genes involved in particular stages of phage defense. Bacterial defense against phages can occur at different stages. In particular, bacteria might prevent adsorption or bacteria might use intracellular defense systems to defend themselves against phage DNA that is already present in the cell. In *P. aeruginosa*, it has been observed that a large proportion of the phages that were not able to infect a particular bacterial strain could still adsorb to its cell surface [7]. If both infection and adsorption data are available, bacterial defense at a particular stage could be analysed with the Goldfinder approach. For example, to infer surface structures involved in adsorption, every phage that can adsorb to a particular bacterium can be encoded as present. Alternatively, to infer intracellular defense systems that prevent infections, only phages that can adsorb but not infect can be encoded as present.

Our approach for identifying candidate genes associated with phage infectivity in bacterial populations comes with a few inherent limitations. First, while large-scale host range matrices are becoming increasingly available owing to automation [18], many studies still provide only limited datasets, with only a few phages or bacterial strains. Our method performs best when at least 20 bacterial strain genomes are available. Additionally, our analysis requires a complete phage–bacteria infection matrix and a discrete classification of phage infectability, which can require setting a manual threshold on continuous measures such as plaque size. The establishment of standardized thresholds and consistent classification systems for phage infectivity [19] would enable the broader application of methods such as the one presented here.

The preliminary analysis of *Vibrio* data also showed that a certain number of interactions must be detected for a phage so that associations can be reconstructed. Nevertheless, our approach also allows researchers to tailor the host range classification to specific biological questions, such as distinguishing between adsorption and infection dynamics.

In addition to these limitations specific to associations with phage infectivity, the Goldfinder approach also comes with general constraints. First, the method relies on accurately reconstructed pangenomes of bacteria and phages and on a bacterial phylogeny. We thus recommend using the latest tools for pangenome reconstruction of bacteria (e.g. Panaroo [9] or panX [10]) and to carefully consider the cutoffs for constructing a phage pangenome. Additionally, to reconstruct an accurate bacterial phylogeny from a core gene alignment, recombination-aware methods could be considered [20]. Second, as the pangenome size increases, so does the number of associations tested, increasing the potential for false positives. Goldfinder corrects for multiple testing based on the false discovery rate, limiting the number of false positives, but not eliminating them. In addition to these randomly distributed false positives, unknown factors that influence gene presence, such as environmental effects, could also result in associations of phage and bacteria genes. Thus, the method can detect direct and indirect effects, and some of the identified interactions in the network will be false positives. Finally, by design, our method detects mobile genes that were gained and lost multiple times along the phylogeny. It thus cannot capture all biological interactions and might also contain false negatives. Particularly, interactions with core genes are not considered by our approach. Additionally, accessory genes that occur only in one or two subclades of the phylogeny will likely not appear in the list of associated pairs, since Goldfinder is designed to focus on associations that are not justifiable owing to common ancestry alone.

In conclusion, our method offers a novel approach for identifying candidate genes within the pool of mobile accessory genes that may contribute to phage–host interactions. It provides a flexible framework applicable to any phage–bacteria system, as long as genetic data are available. While our method may not identify all relevant associations, it provides a statistical approach to uncover patterns that would otherwise require more focused, case-by-case investigations. Thus, the results of the presented approach provide a starting point to design additional experimental and computational approaches to fully resolve the genetic basis of phage susceptibility.

## 4. Technical methods

### (a) Dataset collection and pangenome reconstruction

We use the *Vibrio* dataset from [13], composed of 244 bacteria of the genus *Vibrio* and 227 phages, which infect at least one of these bacteria. These data encompass 1347 positive infections. The assembly fna genome files and gff annotation files were obtained from NCBI. The bacterial pangenome is reconstructed with Panaroo v. 1.5.1 [9] (options –clean-mode strict –remove-invalid-genes), resulting in 27 478 genes in total and 276 soft core genes (present in 95–100% of the strains). The pangenome also includes functional annotation based on the input genome annotations. To detect defense systems in the pangenome, we ran the software DefenseFinder [14] on all bacterial genomes with standard parameters and mapped the identified systems to the genes in the pangenome. Some of the predicted genes in the pangenome cover multiple genes predicted by DefenseFinder. A bacterial strain phylogeny was reconstructed based on the codon alignment of the soft core genes with IQ-TREE v. 2.2.6 (options -B 1000 m GTR+F+I+R10) [11].

### (b) Phage clustering

To estimate the identity between phage genes, we first searched for similarities using blastp v. 2.14.0+ and retained all pairs with an e-value <0.1. For these pairs, global pairwise identity is calculated using Needle from the EMBOSS package v. 6.6.0 [21]. Pairs with identity ≥80% are clustered using mcl v. 14.137 (option -I 2) [22], resulting in 6990 phage gene clusters.

### (c) Goldfinder analysis and network visualization

Goldfinder is a tool designed to detect gene associations in bacterial pangenomes, taking into account the co-occurrence of genetic elements owing to vertical inheritance [8]. In this study, Goldfinder is always run with the options -c both -g 50000 -add 0.999 and otherwise default values. Goldfinder offers a variety of visualization settings to structure the network view, utilizing Cytoscape’s force-directed layout [12]. For example, some visualizations add nodes that represent the centre of clusters of co-occurring genes, and we use a force of 1+ the fraction of associated gene pairs among the two clusters as a means to shape the layout of the network visualization. For example, in figure 2, we only show those clusters where at least 10% of the gene pairs between clusters are significantly associated, which corresponds to a force threshold ≥1.1. This threshold can be manually set by the user to explore the pangenome–phage association network.

## Data Availability

The software Goldfinder and the scripts to generate the Pangenome-Phage-Interaction Matrices (GPAPlusPhage, GPAMinusPhage, and GPAPlusPhageGenes) are available at https://github.com/fbaumdicker/goldfinder.
